# The interplay of comorbidity, disability, and physical activity among older adults living with HIV: insights from the CHANGE HIV study

**DOI:** 10.1186/s12877-025-06939-w

**Published:** 2026-01-06

**Authors:** Tai-Te Su, Kelly K. O’Brien, Alice Zhabokritsky, Bryan Boyachuk, Sharon Walmsley, Correlates of Healthy Aging in Geriatric HIV (CHANGE HIV) study team, G. Arbess, D. Behrens, A. Betts, D. Bowdish, A. Eaton, G. Guaraldi, R. Kaul, J. McCullagh, K. Murzin, R. Newman, P. Rochon, R. Rosenes, G. Sebastiani, A. Tseng, C. Verschoor, C. Wyndham-West

**Affiliations:** 1 Department of Physical Therapy, Temerty Faculty of Medicine, University of Toronto, 160-500 University Avenue, Toronto, ON M5G 1V7 Canada; 2 School and Graduate Institute of Physical Therapy, College of Medicine, National Taiwan University, No 17, Xu-Zhou Road, Taipei, 100 Taiwan; 3 Institute of Health Policy, Management and Evaluation, Dalla Lana School of Public Health, University of Toronto, 155 College Street, Toronto, ON M5T 3M6 Canada; 4 Rehabilitation Sciences Institute, University of Toronto, 160-500 University Avenue, Toronto, ON M5G 1V7 Canada; 5 University Health Network, Immunodeficiency Clinic, 13NU-1300, 585 University Avenue, Toronto, ON M5G 2N2 Canada; 6 Department of Medicine, Division of Infectious Diseases, University of Toronto, 6 Queen’s Park Crescent West, Toronto, ON M5S 3H2 Canada

**Keywords:** HIV, Aging, Comorbidity, Disability, Physical activity

## Abstract

**Background:**

Advances in treatment and care have extended the life expectancy of people living with HIV. Nevertheless, comorbidities are common and may result in health-related challenges, known as disability, in everyday life. Rehabilitation strategies such as physical activity may help to mitigate disability. Our aim was to characterize comorbidity profiles and examine their relationship with disability and physical activity among a cohort of older adults living with HIV in Canada.

**Methods:**

We conducted a cross-sectional analysis of data collected from older adults living with HIV aged 65 years and older and enrolled in the Correlates of Healthy Aging in Geriatric HIV (CHANGE HIV) study. We examined the presence of 14 individual comorbidities and their combinations. Hierarchical linear regression was used to assess the associations between number of comorbidities, disability (Stanford Health Assessment Questionnaire Disability Index), and physical activity (Rapid Assessment of Physical Activity Aerobic Scale) while sequentially adjusting for intrinsic (personal attributes) and extrinsic (perceived HIV stigma and social support) contextual factors.

**Results:**

Among the 516 participants (median age = 69 years, 25th − 75th percentiles: 67–73), most were identified as male (90%) and White (77%). Participants reported a median of two comorbidities (25th − 75th percentiles: 1–4) in addition to HIV. The most common comorbidities included dyslipidemia (51%), hypertension (45%), cancer (28%), diabetes (23%), and arthritis (21%). Various combinations of coexisting comorbidities were also observed. A greater number of comorbidities was associated with more severe disability scores (*ρ* = 0.25, *p* < 0.001). However, higher levels of physical activity attenuated the impact of each additional comorbidity on disability scores, and this moderating effect remained robust after accounting for the influence of intrinsic and extrinsic contextual factors.

**Conclusions:**

Comorbidities are prevalent among older adults living with HIV in Canada and are associated with disability. Physical activity attenuated the negative association between comorbidity and disability, highlighting that older adults living with HIV who are physically active may experience better functional outcomes. Routine screening and management of chronic conditions, coupled with tailored physical activity interventions, may have a role in addressing disability among older adults living with HIV.

**Trial registration:**

Clinical trial not applicable.

**Supplementary Information:**

The online version contains supplementary material available at 10.1186/s12877-025-06939-w.

## Background

Advances in antiretroviral therapy and medical care in the context of HIV have dramatically improved survival, allowing more people living with HIV to live longer [1]. Using population-based data from the Netherlands, a simulation study estimated that 28% of people living with HIV were aged 50 years or older in 2010, with this proportion projected to increase to 73% by 2030, and nearly 40% expected to be aged 60 years or older by that time [2]. In Canada, similarly, national HIV surveillance data from the Public Health Agency of Canada showed that the proportion of newly diagnosed HIV cases among adults aged 50 years or older increased from 15.1% in 2008 to 22.8% in 2017 [3]. Additionally, one in six of all people living with HIV are projected to be over 50 years of age by 2025 [4]. Overall, the demographic shift toward an aging population of people living with HIV is expected to occur worldwide [2, 5].

In tandem with these successes in HIV care, a central issue accompanying longer lifespan is the presence of concurrent health conditions in addition to HIV, commonly referred to as comorbidities [6, 7]. Compared to the general population, people living with HIV are more likely to develop physical comorbidities such as hypertension, myocardial infarction, peripheral vascular disease, and chronic kidney disease [8], as well as mental or psychological comorbidities including depression, psychological distress, and post-traumatic stress disorder that may emerge or persist across the lifespan [9]. The presence of physical comorbidities may reflect in part the long-term use of antiretroviral therapy and persistent immune activation, with a greater burden observed among individuals with higher cumulative viremia [10–12]. For example, exposure to older antiretroviral agents such as stavudine and didanosine has been associated with the development of peripheral neuropathy [13]. Additionally, individuals with intermittent antiretroviral use have been shown to experience a higher burden of comorbidities compared with those maintaining continuous antiretroviral therapy and viral suppression [14]. Population-based analyses using the Ontario Health Insurance Plan (OHIP) data showed that approximately 35% of people living with HIV had at least one other physical comorbidity (e.g., congestive heart failure and chronic obstructive pulmonary disease), and that the prevalence of multimorbidity (defined as living with two or more comorbidities) increased with age across both men and women [15]. These findings highlight that comorbidity is not only prevalent but represents a significant challenge of aging with HIV.

In the context of comorbidity, older adults living with HIV may also experience disability in everyday life. Notably, the definition of disability varies across fields. In rehabilitation or aging research, disability is commonly described as difficulty or dependency in carrying out daily activities within a given environment [16]. In the HIV literature, disability is conceptualized more broadly as health-related challenges arising from the direct and indirect effects of HIV, its secondary conditions, and the side effects of medications [17, 18]. Regardless of its definition, the experience of disability reflects functional limitations and everyday challenges, and it is closely linked to other adverse outcomes such as frailty, mortality, and reduced quality of life for persons aging with HIV [19]. Preventing or mitigating disability is therefore a critical goal in supporting healthy aging with HIV.

Physical activity represents a key nonpharmacological rehabilitation strategy and plays an important role in maintaining the health and function of people living with HIV. A growing body of evidence indicates that physical activity and structured exercise interventions are safe and beneficial for people living with HIV, demonstrating improvements in physical capacity, cardiorespiratory fitness, neurocognitive function, metabolic outcomes, and overall quality of life [20–24]. Although the benefits of physical activity are well established, most research to date has focused on younger or middle-aged adults, and evidence regarding its effects in older adults living with HIV is still emerging [25]. Additionally, there remains a lack of research examining the role of physical activity in the context of comorbidity and disability specifically among older adults living with HIV. Given the increasing number of older adults living with HIV, there is an urgent need to better understand how comorbidity, disability, and physical activity intersect in later life. Our objectives of this study were threefold: (1) to characterize the profile of comorbidity among older adults living with HIV; (2) to assess the association between comorbidity and disability; and (3) to examine whether physical activity moderates the relationship between comorbidity and disability among a Canadian cohort of people living with HIV aged 65 years and older.

## Methods

### Study design and participants

We conducted a quantitative, cross-sectional analysis of data collected from the Correlates of Healthy Aging in Geriatric HIV (CHANGE HIV) study [26]. Established in 2019, the CHANGE HIV study represents the first prospective cohort of people living with HIV aged 65 years and older in Canada. The overarching aim of the CHANGE HIV study is to advance knowledge in HIV and aging by characterizing the multidimensional health status of older adults living with HIV in Canada and identifying determinants of healthy aging [26]. To be eligible for inclusion, participants needed to be diagnosed with HIV and aged 65 years and older, actively receiving HIV care at one of the seven participating sites across three Canadian provinces (British Columbia, Ontario, and Quebec), and having the ability to speak and understand English or French to complete study procedures. Detailed information on participant enrollment is documented in the parent study description [26]. In the current study, our final analytic sample was restricted to CHANGE HIV participants who responded to the comorbidity, disability, and physical activity questionnaires during the baseline assessment, which are the primary foci of the present study. The CHANGE HIV study received approval from the Research Ethics Board (REB) from the University Health Network (CAPCR 18–6311) and participating sites. All participants provided written informed consent prior to any study activities. The current study received ethics approval from the Health Sciences REB at the University of Toronto (protocol # 47106).

### Measures

#### Comorbidity

 We defined comorbidity as the the presence of concurrent health conditions in addition to HIV identified by chart review or self-report from the following 14 conditions: hypertension, coronary heart disease, congestive heart failure, dyslipidemia, stroke, peripheral neuropathy, Parkinson’s disease, chronic obstructive pulmonary disease, osteoporosis, arthritis, diabetes, chronic kidney disease, cancer, and depression. The aforementioned comorbidities were selected as they have been commonly reported among older adults living with HIV [6, 9, 26] and/or included in the construction of the Rotterdam Healthy Aging Score [27] used to measure healthy aging in the cohort. Final comorbidity scores reflect the total number of comorbidities (range: 0–14). For descriptive purposes, participants were categorized as living with multimorbidity (≥ 2 comorbidities) or without multimorbidity (≤ 1 comorbidity).

#### Disability

We operationally defined disability as difficulty performing day-to-day activities, which was measured using the Stanford Health Assessment Questionnaire Disability Index (HAQ-DI) [28, 29]. During the questionnaire assessment, participants self-reported their functional ability with respect to eight basic activities of daily living (ADLs), including dressing and grooming, arising, eating, walking, hygiene, reach, grip, and daily activities. Responses were rated on a 4-point ordinal scale ranging from 0 (*without any difficulty*) to 3 (*unable to do*). The eight ADLs scores were averaged into an overall HAQ-DI score ranging from 0 to 3, with higher scores indicating more severe disability. Although the HAQ was originally developed in the field of rheumatology, it was not designed as a disease-specific instrument but rather a generic patient-oriented outcome assessment tool [28]. The HAQ demonstrates reliability and validity and has been applied in studies with various populations such as people living with HIV, general aging populations, and people living with rheumatic diseases [29–33].

#### Physical activity

 Physical activity was assessed using the aerobic scale of the Rapid Assessment of Physical Activity (RAPA) [34]. Developed based on the Centers for Disease Control and Prevention (CDC) physical activity guidelines, the RAPA is a self-reported questionnaire that measures the frequency and intensity of moderate and vigorous activity. Participants were classified into five increasing levels of physical activity: (Level 1) *Sedentary*: rarely or never do any physical activities; (Level 2) *Underactive*: do some light or moderate physical activities, but not every week; (Level 3) *Underactive regular light*: do some light physical activity every week; (Level 4) *Underactive regular*: do moderate activity weekly but < 30 min/day or < 5 days/week, or do vigorous activity weekly but < 20 min/day or < 3 days/week; (Level 5) *Active*: do ≥ 30 min/day of moderate activity on ≥ 5 days/week, or ≥ 20 min/day of vigorous activity on ≥ 3 days/week. The RAPA demonstrates construct validity and responsiveness among adults living with HIV [35].

#### Contextual factors

Guided by the Episodic Disability Framework and literature [17, 36], we included a number of intrinsic (personal) and extrinsic contextual factors that may have affected the interplay of comorbidity, disability, and physical activity. The terms (intrinsic and extrinsic factors) are used within the Framework to describe features or factors that may exacerbate or alleviate disability among people living with HIV. Intrinsic contextual factors encompassed personal attributes and health-related factors. Personal attributes including age, sex, race/ethnicity, educational attainment, gross annual household income, marital status, and employment status were measured at baseline using a sociodemographic questionnaire. For health-related factors, we included information on participants’ CD4^+^ nadir (cells/mm^3^) and age at time of HIV diagnosis (< 50 years or ≥ 50 years). In addition, we measured physical capacity using the Short Physical Performance Battery (SPPB) Score, which involved objective assessments of balance, gait, and chair stands [37]. Each component was scored from 0 to 4, with higher scores indicating better performance. Moreover, we assessed the level of loneliness using the 20-item UCLA Loneliness Scale [38]. Final scores ranged from 20 to 80, with higher scores suggesting higher degree of loneliness. For extrinsic contextual factors, we assessed the extent of HIV stigma using the 12-item HIV Stigma Scale (range: 12–60) and perceived social support using the 19-item RAND Social Support Survey (range: 0–100) [39, 40]. Higher scores reflect greater HIV stigma and greater social support, respectively. Both instruments have demonstrated reliability and validity among people living with HIV [39, 41, 42].

### Statistical analysis

We conducted descriptive statistics to summarize participants’ characteristics. Medians and 25th and 75th percentiles were reported for continuous variables, and counts and percentages were reported for categorical variables. Next, we constructed Upset plots to characterize the presence of each individual comorbidity and the most frequently observed comorbidity combinations. Spearman’s rank correlation coefficient (*ρ*) was calculated to quantify the association between number of comorbidities and disability severity. The correlation coefficients were considered negligible (*ρ* < 0.10), weak (0.10 ≤ *ρ* < 0.40), moderate (0.40 ≤ *ρ* < 0.70), or strong (*ρ ≥* 0.70) [43]. As an exploratory step, we conducted Wilcoxon rank sum tests and chi-squared tests to examine whether personal characteristics differed between participants living with and without multimorbidity (i.e., living with ≥ 2 comorbidities). All analyses were conducted using R statistical software (version 4.3.1; R Core Team, 2023).

To formally assess the relationships between comorbidity, disability, and physical activity, we applied hierarchical linear regression and incorporated contextual factors sequentially in blocks. In other words, we started with a model containing only the primary variables of interest and their interaction, and then introduced groups of contextual factors in a stepwise manner to determine whether the observed associations remained robust after adjustment. In Model 1 (the base model), we specified disability as the dependent variable and regressed it on comorbidity, physical activity, and their interaction. This allowed us to examine the main effects of comorbidity and physical activity on disability, as well as whether the association between comorbidity and disability varied across different levels of physical activity. Model 2 retained the variables in Model 1 and additionally included contextual factors related to personal attributes. Model 3 built on Model 2 and included health-related factors. Model 4 included all previous variables and added HIV stigma and perceived social support to capture the broader contextual influence. Missing data on contextual factors was assumed to be missing at random and addressed using multiple imputations (m = 10) to maintain the maximum possible sample size. The significance level for statistical analyses was set at *p* < 0.05.

## Results

### Participant characteristics

Between October 10, 2019 and March 27, 2025, a total of 550 participants were enrolled in the CHANGE HIV study. Among these participants, 516 (93.8%) responded to the comorbidity, disability, and physical activity questionnaires during the baseline assessment and were included in the analyses. Table 1 summarizes the sociodemographic and health-related characteristics of the entire sample as well as stratified by multimorbidity status. The median age of all participants was 69 years (25th − 75th percentiles: 67–73). Most participants were male (90%) and identified as White (77%). The most common education level was having a bachelor’s degree or higher (41%), whereas over half of the participants reported a gross household income below $50,000 CAD (55%). Participants reported a median of two comorbidities (25th − 75th percentiles: 1–4) in addition to HIV. The median disability severity score, as measured by the HAQ-DI, was 0 (mean = 0.18; standard deviation ± 0.40). With respect to physical activity levels, 4% were considered *sedentary* (Level 1) while 58% were classified as *active* (Level 5).

**Table 1:** Baseline characteristics of the CHANGE HIV participants by multimorbidity status

Participant characteristics	Pooled (*n* = 516)	With multimorbidity* (*n* = 371)	Without multimorbidity (*n* = 145)	*p*-value
	Median [25th, 75th percentiles]/*n* (%)			
Comorbidity (counts)	2 [1, 4]	3 [2, 4]	1 [0, 1]	< 0.001
Age (years)	69 [67, 73]	69 [67, 73]	68 [66, 72]	0.041
Male sex, n (%)	463 (90)	336 (91)	127 (89)	0.666
Race/Ethnicity, n (%)				0.331
White	395 (77)	292 (79)	103 (73)	
Black	64 (12)	43 (12)	21 (15)	
Other (Asian, Hispanic, Aboriginal, etc.)	54 (11)	36 (10)	18 (13)	
Education				0.342
Trades certificate or below	159 (31)	115 (31)	44 (31)	
College or university diploma	140 (27)	95 (26)	45 (32)	
Bachelor’s degree or higher	212 (41)	159 (43)	53 (37)	
Household income (CAD)				0.333
Under $50,000	278 (55)	208 (57)	70 (50)	
$50,000 – $99,999	136 (27)	95 (26)	41 (29)	
More than $100,000	90 (18)	61 (17)	29 (21)	
Marital status, n (%)				0.038
Unpartnered	337 (66)	254 (69)	83 (58)	
In a relationship	175 (34)	116 (31)	59 (42)	
Employment, n (%)				0.103
Retired	374 (73)	280 (75)	94 (66)	
Employed (full time or part time)	89 (17)	59 (16)	30 (21)	
Other (volunteer, homemaker, etc.)	50 (10)	32 (9)	18 (13)	
Age group at HIV diagnosis				0.331
Younger than 50 years	349 (71)	257 (73)	92 (68)	
50 years and older	141 (29)	97 (27)	44 (32)	
CD4^+^ nadir, cells/mm^3^	200 [96, 340]	190 [85, 310]	244 [130, 380]	0.024
Short Physical Performance Battery score (SPPB; 0–12)	9 [8, 11]	9 [8, 11]	9 [8, 11]	0.628
Balance score (0–4) †	4 [4, 4]	4 [4, 4]	4 [4, 4]	0.029
Gait score (0–4)	4 [2, 4]	4 [2, 4]	4 [2, 4]	0.989
Chair stand score (0–4)	2 [1, 4]	2 [1, 4]	2 [1, 3]	0.815
Loneliness score (20–80)	27 [22, 41]	28 [22, 41]	25 [21, 40]	0.150
HIV Stigma score (12–60)	25 [20, 30]	26 [20, 30]	25 [21, 30]	0.982
Social support total score (0–100)	67 [47, 87]	67 [47, 85]	71 [47, 88]	0.264
Emotional/information support (8–40)	31 [24, 37]	31 [24, 37]	31 [24, 36]	0.802
Tangible support (4–20)	16 [10, 20]	15 [9, 20]	16 [10, 20]	0.196
Affectionate support (3–15)	12 [7, 15]	11 [7, 14]	12 [8, 15]	0.127
Positive social interaction (4–20)	16 [12, 20]	15 [12, 19]	16 [12, 20]	0.041
Disability score (HAQ-DI) (0–3)	0 [0, 0.3]	0 [0, 0.3]	0 [0, 0]	< 0.001‡
Physical activity level				0.401
Level 1: Sedentary	22 (4)	18 (5)	4 (3)	
Level 2: Underactive	23 (4)	18 (5)	5 (3)	
Level 3: Underactive regular light	53 (10)	40 (11)	13 (9)	
Level 4: Underactive regular	121 (23)	91 (25)	30 (21)	
Level 5: Active	297 (58)	204 (55)	93 (64)	

*Multimorbidity is defined as having two or more comorbidities in addition to living with HIV. † The mean ± standard deviation balance score was 3.65 ± 0.82 for participants with multimorbidity and 3.80 ± 0.64 for those without multimorbidity. ‡ The mean ± standard deviation disability severity was 0.22 ± 0.42 for participants living with multimorbidity and 0.11 ± 0.32 for those without multimorbidity. HAQ-DI: Health Assessment Questionnaire Disability Index

### Presence of comorbidities and associated findings

**Fig. 1:**
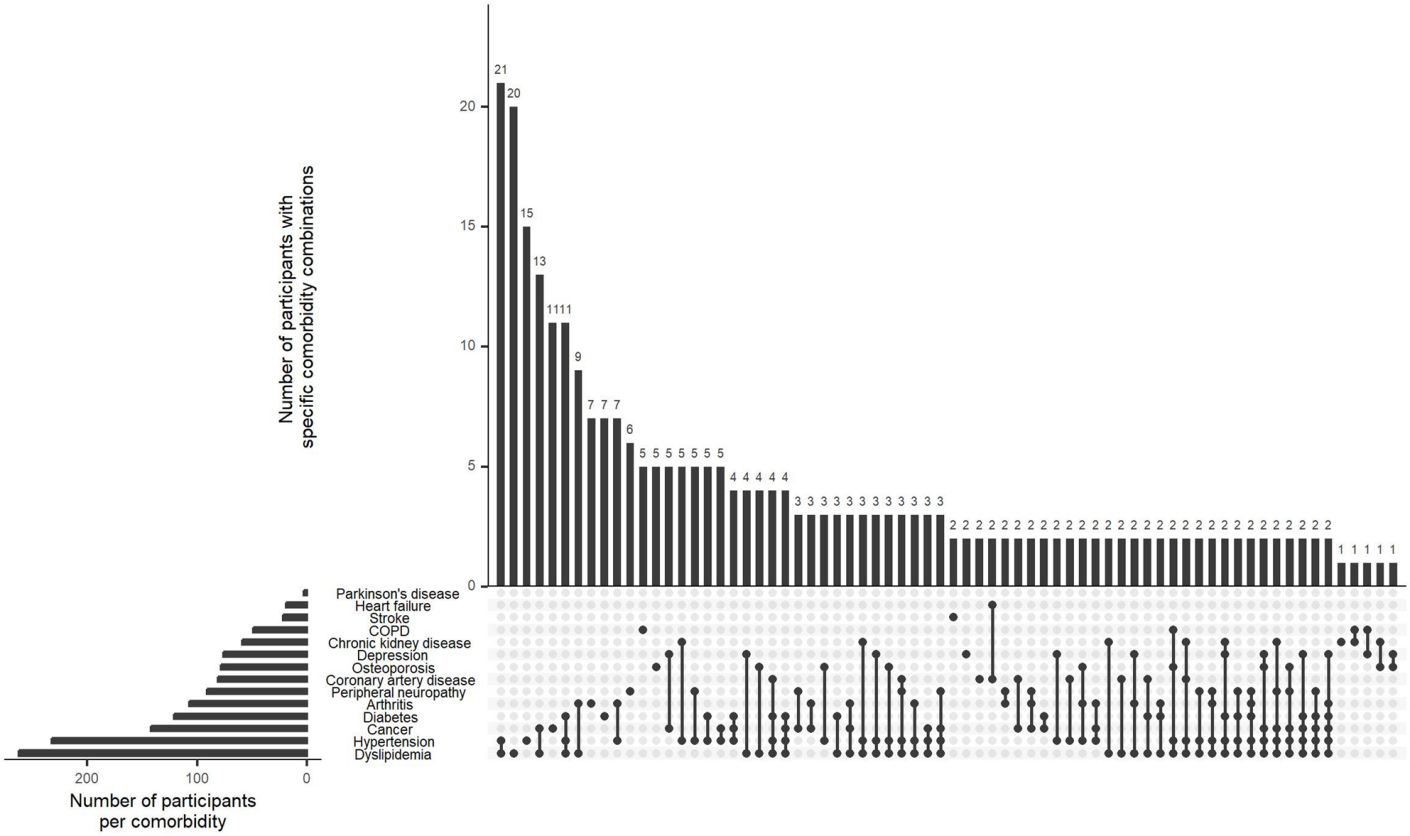
Presence and most common combinations of comorbidities among older adults living with HIV in the CHANGE HIV study. Note: The UpSet plots visually illustrate both the presence of each individual comorbidity (displayed in the horizontal bar plot) and the combinations of these comorbidities (displayed in the vertical bar plot). For instance, dyslipidemia is the most frequently reported comorbidity, while the most common combination involves the co-occurrence of dyslipidemia and hypertension. Moreover, the combinations of comorbidities were in fact diverse and did not follow a clear pattern, underscoring the complex health challenges experienced among older adults living with HIV

**Fig. 2:**
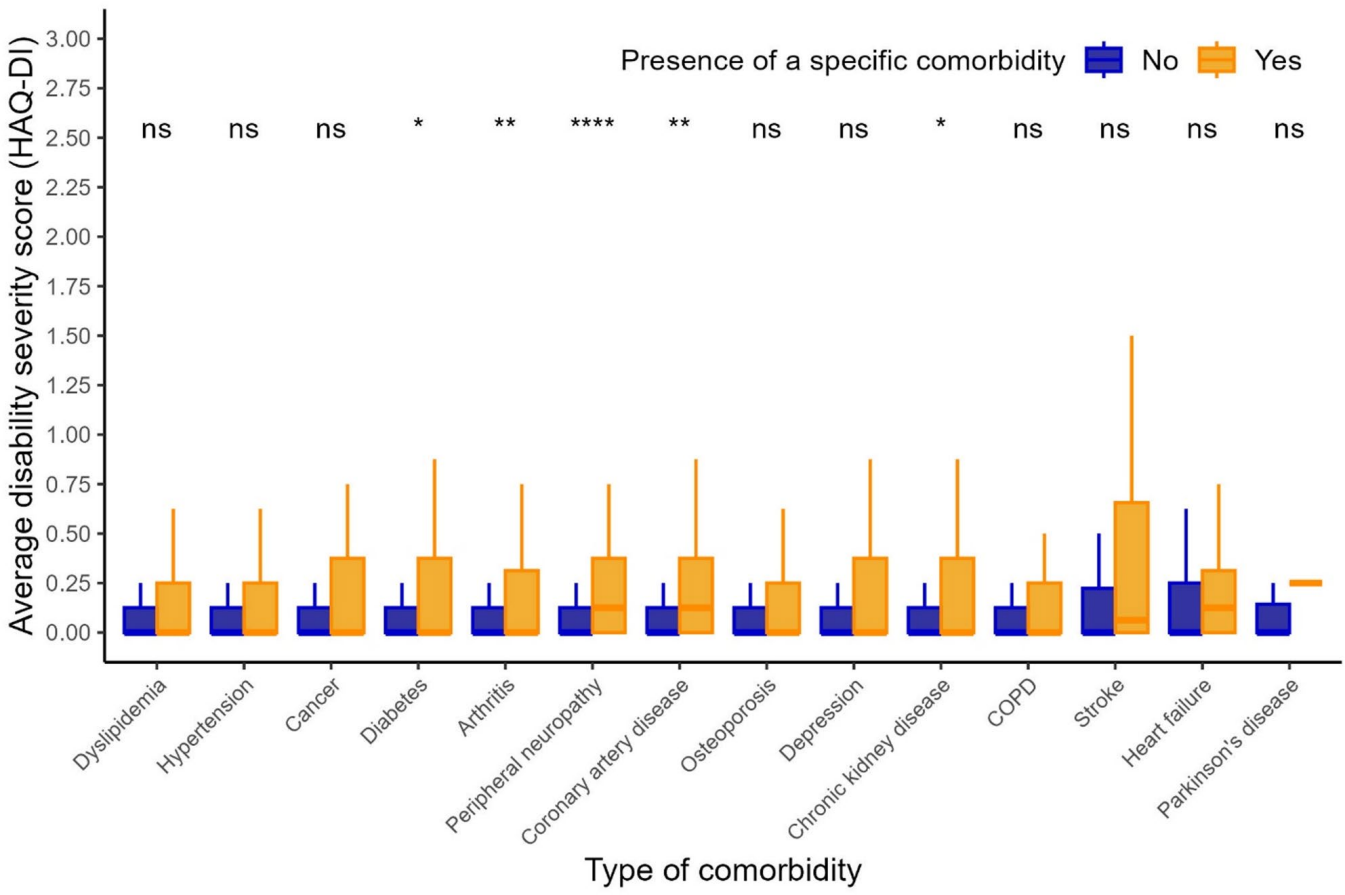
Distribution of disability severity scores as measured by the HAQ-DI by presence or absence of individual comorbidities. Note: Wilcoxon rank sum tests were used to compare disability severity scores between participants living with and without each individual comorbidity. ns: *p*> 0.05. *: *p* <= 0.05; **: *p* <= 0.01; ***: *p* <= 0.001; ****: *p* <= 0.0001; COPD: chronic obstructive pulmonary disease

**Fig. 3:**
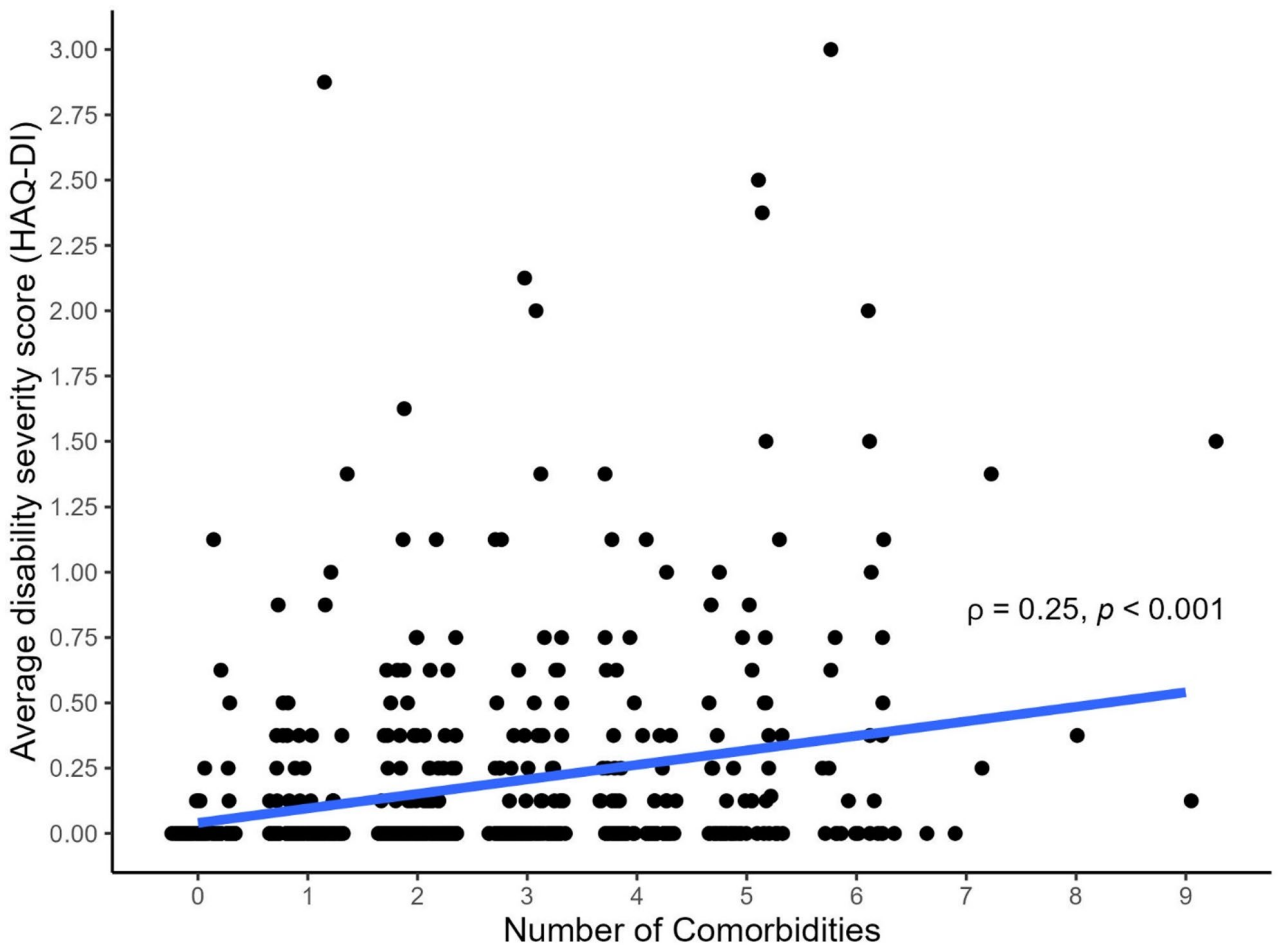
Correlation between number of comorbidities and disability severity score (*n* = 516). Note: Spearman’s rank correlation coefficient (*ρ*) was calculated to quantify the association between comorbidity and disability. The correlation coefficients were interpreted as negligible (*ρ* < 0.10), weak (0.10 ≤ *ρ* < 0.40), moderate (0.40 ≤ *ρ* < 0.70), or strong (*ρ* ≥ 0.70)

Overall, 371 (72%) participants were living with multimorbidity (defined as living with ≥ 2 comorbidities; Table 1). Participants living with multimorbidity were, on average, older, less likely to be partnered, had lower nadir CD4 count, lower balance scores, reported less positive social interaction, and experienced greater disability compared to those living without multimorbidity (Table 1). The most frequently reported types of comorbidities were dyslipidemia (51%), hypertension (45%), cancer (28%), diabetes mellitus (23%), and arthritis (21%) (Table 2). This pattern was consistent regardless of multimorbidity status. Figure 1 visually illustrates the presence and combinations of the 14 comorbidities under investigation. Results showed that dyslipidemia and hypertension were the most commonly co-occurring conditions, while a wide variety of other comorbidity combinations were also observed. Regarding the bivariate associations between comorbidities and disability, diabetes, arthritis, peripheral neuropathy, coronary artery disease, and chronic kidney disease were each significantly associated with higher disability scores (Fig. 2). Moreover, there was a weak but significant positive correlation between the number of comorbidities and disability severity (Spearman’s rank correlation coefficient *ρ* = 0.25, *p* < 0.001; Fig. 3). Cross-tabulations of each comorbidity by physical activity level are presented in Supplementary Table 1.

**Table 2:** Most frequently reported type of comorbidities among the CHANGE HIV participants

Frequency of Comorbidity	Pooled (*n* = 516)	With multimorbidity (*n* = 371)	Without multimorbidity (*n* = 145)
		Count (%)	
Most frequent	Dyslipidemia	Dyslipidemia	Dyslipidemia
	262 (51%)	242 (65%)	20 (14%)
Second most frequent	Hypertension	Hypertension	Hypertension
	232 (45%)	217 (58%)	15 (10%)
Third most frequent	Cancer	Cancer	Cancer
	142 (28%)	131 (35%)	11 (8%)
Fourth most frequent	Diabetes	Diabetes	Diabetes
	121 (23%)	114 (31%)	7 (5%)
Fifth most frequent	Arthritis	Arthritis	Arthritis
	107 (21%)	100 (27%)	7 (5%)

### Relationships between comorbidity, disability, and physical activity

**Fig. 4:**
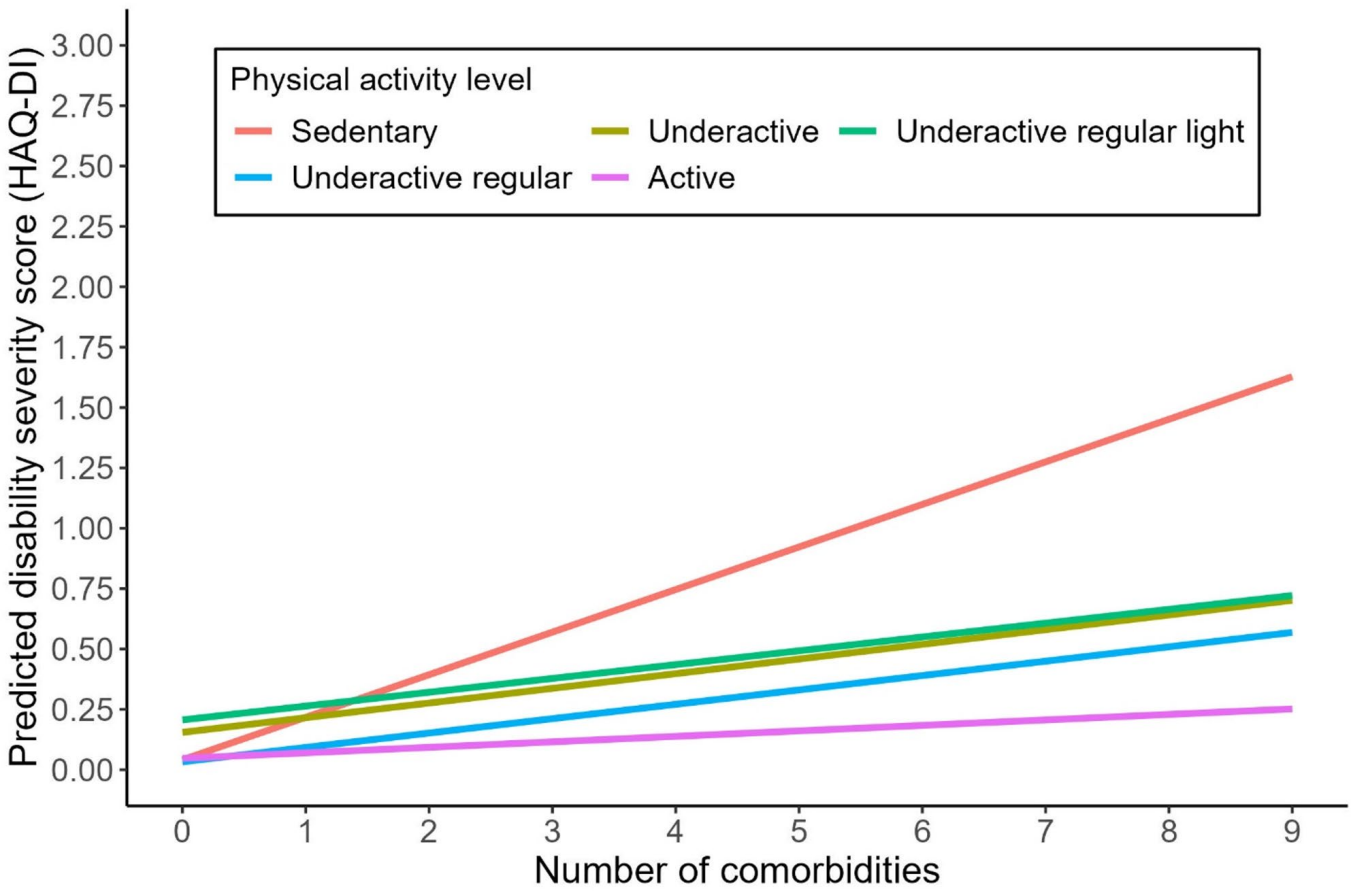
The moderating role of physical activity in the relationship between comorbidity and disability. Note: The interaction plot illustrates the moderating role of physical activity in the base model (Model 1 in Table 3). Specifically, higher physical activity levels attenuate the impact of each additional comorbidity on disability severity among older adults living with HIV. This moderating pattern remains consistent after adjusting for contextual factors (Models 2–4 in Table 3)

Table 3 summarizes the results of hierarchical linear regression models assessing the associations between comorbidity, disability, and physical activity. In Model 1 (base model), a greater number of comorbidities was associated with more severe disability (*b* = 0.18, 95% CI: 0.09, 026, *p* < 0.001). There was no significant direct association between physical activity level and disability severity. However, statistically significant comorbidity × physical activity interactions were observed, indicating that higher levels of physical activity significantly moderated the impact of comorbidity on disability. To be more specific, each additional comorbidity was associated with a 0.18-point increase in disability severity among participants who were sedentary (Level 1). Nevertheless, this effect was attenuated to 0.06 points (*b* = 0.18 − 0.12) among those who were underactive (Level 2), and further reduced to 0.03 points (*b* = 0.18 − 0.15) among those who were active (Level 5), suggesting a potential dose-response effect (Fig. 4). The moderating role of physical activity in the relationship between comorbidity and disability remained robust across models that accounted for the influence of intrinsic and extrinsic contextual factors. In Model 4 (fully adjusted model), physical capacity as measured by the SPPB was the only significant contextual factor associated with disability in addition to comorbidities. All included factors explained 31% of the total variance in disability.

**Table 3:** Hierarchical associations between comorbidity, disability, and physical activity, adjusting sequentially for contextual factors

Contextual factors	Outcome: severity of disability (range: 0–3)			
	Model 1	Model 2	Model 3	Model 4
	Unstandardized regression coefficients *Beta* [95% confidence intervals]			
Comorbidity (counts)	0.18 [0.09, 026]***	0.18 [0.10, 0.27]***	0.15 [0.07, 0.23]***	0.15 [0.07, 0.23]***
Physical activity level (ref: sedentary)				
Underactive	0.11 [–0.31, 0.54]	0.16 [–0.26, 0.58]	0.04 [–0.35, 0.43]	0.02 [–0.38, 0.41]
Underactive regular light	0.17 [–0.21, 0.54]	0.22 [–0.16, 0.59]	0.24 [–0.11, 0.58]	0.22 [–0.13, 0.56]
Underactive regular	–0.01 [–0.36, 0.34]	0.02 [–0.33, 0.37]	0.01 [–0.32, 0.34]	–0.00 [–0.33, 0.33]
Active	0.01 [–0.33, 0.34]	0.05 [–0.29, 0.39]	0.07 [–0.24, 0.39]	0.05 [–0.26, 0.37]
Comorbidity × Underactive	–0.12 [–0.23, − 0.00]*	–0.13 [–0.24, − 0.02]*	–0.10 [–0.21, − 0.00]*	–0.10 [–0.20, 0.00]
Comorbidity × Underactive regular light	–0.12 [–0.22, − 0.02]*	–0.14 [–0.23, − 0.04]**	–0.13 [–0.22, − 0.04]**	–0.12 [–0.21, − 0.03]**
Comorbidity × Underactive regular	–0.12 [–0.21, − 0.02]*	–0.13 [–0.22, − 0.03]**	–0.10 [–0.19, − 0.01]*	–0.10 [–0.19, − 0.01]*
Comorbidity × Active	–0.15 [–0.24, − 0.06]***	–0.16 [–0.25, − 0.08]***	–0.14 [–0.22, − 0.06]**	–0.13 [–0.22, − 0.05]**
Age (per 10-year)		0.03 [–0.05, 0.10]	0.02 [–0.05, 0.09]	0.03 [–0.05, 0.10]
Sex (ref: female)				
Male		–0.05 [–0.16, 0.06]	–0.03 [–0.13, 0.07]	–0.03 [–0.13, 0.08]
Race/Ethnicity (ref: White)				
Black		–0.02 [–0.12, 0.09]	–0.01 [–0.11, 0.09]	–0.05 [–0.15, 0.06]
Other		0.09 [–0.01, 0.19]	0.11 [0.01, 0.20]*	0.09 [–0.00, 0.19]
Education (ref: trades certificate or below)				
College or university diploma		0.04 [–0.04, 0.13]	0.04 [–0.04, 0.12]	0.05 [–0.03, 0.12]
Bachelor’s degree or higher		0.07 [–0.01, 0.15]	0.05 [–0.03, 0.13]	0.05 [–0.02, 0.13]
Household income (ref: under $50,000)				
$50,000 – $99,999		–0.09 [–0.17, − 0.01]*	–0.07 [–0.14, 0.01]	–0.07 [–0.14, 0.00]
More than $100,000		–0.07 [–0.17, 0.03]	–0.04 [–0.13, 0.06]	–0.04 [–0.14, 0.05]
Marital status (ref: unpartnered)				
In a relationship		–0.04 [–0.11, 0.04]	0.00 [–0.07, 0.07]	–0.01 [–0.08, 0.07]
Employment (ref: retired)				
Employed		–0.09 [–0.18, − 0.01]*	–0.06 [–0.15, 0.02]	–0.07 [–0.15, 0.02]
Other (volunteer, homemaker, etc.)		–0.06 [–0.17, 0.05]	–0.04 [–0.14, 0.06]	–0.03 [–0.13, 0.07]
Age group at HIV diagnosis (ref: <50 years)				
50 years and older			–0.02 **[**–0.09, 0.06**]**	–0.03 [–0.10, 0.04]
CD4^+^ nadir (per 100 cells/mm^3^)			–0.00 [–0.02, 0.01]	–0.00 [–0.02, 0.01]
Physical capacity (SPPB score)			–0.07 [–0.08, − 0.05]***	–0.07 [–0.08, − 0.05]***
Loneliness (per 10-point)			0.03 [0.00, 0.06]*	0.03 [–0.00, 0.06]
HIV stigma (per 10-point)				0.05 [–0.01, 0.11]
Social support (per 10-point)				0.01 [–0.01, 0.02]
Adjusted R^2^	0.16 [0.11, 0.22]	0.18 [0.12, 0.24]	0.31 [0.24, 0.38]	0.31 [0.25, 0.38]

Ref = reference group. Model 1 tested the main and interaction effects of comorbidity and physical activity on disability severity. Model 2 included personal characteristics and socioeconomic status. Model 3 included correlates in Model 2 plus HIV-specific metrics and health-related factors. Model 4 included correlates in Model 3 plus perceived stigma and social support. Unstandardized regression coefficients were reported * *p*<.05. ** *p*<.01. *** *p*<.001

Recognizing that only a small proportion of older adults living with HIV was sedentary (*n* = 22; 4%), we performed sensitivity analysis and reran the models described above, in which participants at RAPA Levels 1 (*sedentary*) through 4 (*underactive regular*) were combined into a single non-active group (*n* = 219; 42%) and compared with those at Level 5 (*active*) to test the robustness of our findings. The overall patterns were consistent with the primary analyses and are reported in Supplementary Tables 2 and Supplementary Fig. 1.

## Discussion

In this study, we characterized the profile of comorbidities and investigated their relationships with disability and physical activity among a sample of older adults (≥ 65 years age) living with HIV in Canada. We observed that 72% of the participants were living with two or more comorbidities in addition to their HIV, and this was higher among those reporting a lower nadir CD4 count. Overall, 58% of participants reported being active with only 4% sedentary. Our findings showed that the number of comorbidities was significantly associated with greater disability scores. Meanwhile, physical activity served as a protective factor, mitigating the influence of comorbidity on disability in the context of aging with HIV.

Overall, we found that dyslipidemia, hypertension, and cancer emerged as the three most commonly reported comorbidities among this sample of older adults living with HIV in Canada. This pattern was consistent with findings from early analyses of the CHANGE HIV cohort and aligned with the broader literature on HIV and aging, where cardiometabolic diseases and non-AIDS-defining cancers (e.g., lung, liver, anal cancers) were frequently documented among older adults living with HIV [7, 8, 44]. Although HIV is now widely considered a chronic, manageable condition for those with access to antiretroviral therapy, the presence of other concurrent health conditions reflects the natural aging process as well as the potential contributions of persistent immune activation, chronic inflammation, toxicity, and adverse metabolic effects associated with HIV and long-term use of antiretroviral therapies [7, 45, 46]. Moreover, while estimates of multimorbidity may not be directly comparable across studies due to differences in the number and type of conditions included, the high prevalence of multimorbidity (72%) observed in this study is similar to that reported in the AGE_h_IV Cohort Study in the Netherlands (70%) [8], underscoring the unique, compounded challenges experienced by people aging with HIV compared to the general older population. In addition to routine HIV care, our findings underscore the need for comprehensive geriatric assessment and management strategies with special attention to issues such as cardiovascular risk assessment, cancer screening, and polypharmacy among older adults living with HIV [47–49].

The mean disability severity score, as measured by the HAQ-DI, was 0.18 (on a 0–3 scale) in this study. Notably, this mean score was lower than values reported for people living with osteoarthritis (mean = 0.80) or rheumatoid arthritis (mean = 1.20) using the same instrument [28]. At first glance, this finding may seem counterintuitive given the health-related challenges reported by people living with HIV. However, several considerations may help explain this discrepancy. First, although the HAQ-DI is widely used to assess disability, prior studies have identified potential floor effect and reduced sensitivity among people with high levels of physical functioning [50–52]. A similar issue was reported by Brañas and colleagues [53], who found that older adults living with HIV could achieve full scores on the Barthel Index (BI) and Functional Ambulation Classification (FAC) despite having underlying impairments or frailty. Second, the HAQ-DI emphasizes limitations in performing basic ADLs, which may underrepresent other disability domains that are relevant to aging with HIV [17]. For example, recent studies using the HIV Disability Questionnaire (HDQ) and the Episodic Disability Questionnaire (EDQ), which both capture the multidimensional nature of disability among people living with HIV, showed that disability scores were lowest in the domain of difficulty with day-to-day activities, while higher levels of disability were observed in domains such as mental or emotional symptoms and impairments, uncertainty or worry about the future, and challenges to social inclusion [54, 55]. Our findings also may reflect the nature of recruitment for the CHANGE HIV study, which includes community-dwelling older adults receiving HIV care. Despite the low average score and unidimensional focus of the HAQ-DI, it is noteworthy that our findings still demonstrated a clear gradient, as participants living with a greater number of comorbidities experienced more severe disability. These findings highlighted that comorbidities, if left unattended, can compromise functional independence, which in turn may contribute to other adverse outcomes such as frailty, hospitalization, and mortality in older adults living with HIV.

Our study found that physical activity significantly moderated the relationship between comorbidity and disability among older adults living with HIV. Specifically, higher levels of physical activity attenuated the negative impact of comorbidity on disability, and this moderating effect remained robust even after adjustments for intrinsic and extrinsic contextual factors. While we were unable to strictly determine the direction of causality, the present study builds on the extant body of literature and reinforces the well-established role of regular physical activity and exercise in improving strength, fitness, body composition, neurocognitive functioning, and overall quality of life in people living with HIV [22, 56–58]. Additionally, exercise and physical activity have been shown to improve vascular aging in people living with HIV and to produce clinically important reductions in psychological distress among older adults with subthreshold depression or anxiety [59, 60]. Given the documented benefits of physical activity, the critical question now is how to support engagement in physical activity among older adults living with HIV. To answer this question, it is essential to first recognize the barriers that may hinder participation. In the general older adult population, common barriers include physical limitations, pain, lack of professional guidance, unsupportive environments, and socio-cultural stereotypes associated with aging [61, 62]. Older adults living with HIV face these same challenges and may encounter additional barriers to engaging in physical activity such as HIV stigma and fear of inadvertent HIV status disclosure accessing traditional gym environments [63–65]. Although examining these factors was beyond the scope of the current study, our findings underscore the importance of creating supportive environments and infrastructures that facilitate sustained physical activity engagement for this population. Ideally, efforts to promote physical activity should begin earlier in life to establish habits that can be maintained through aging. Additionally, intervention programs and rehabilitation and health providers should take into account the varying goals, preferences, and needs of older adults living with HIV, particularly those who are interested in physical activity but may experience complex life situations and unpredictable changes in health status [66]. In the meantime, technology-mediated solutions such as use of wireless physical activity monitors, online coaching, and virtual home-based exercise programs may serve as a promising avenue to enhance engagement, monitoring, and adherence to physical activity among older adults living with HIV [67, 68].

To our knowledge, this study is among the first to characterize the intertwined relationships between comorbidity, disability, and physical activity specifically among people aged 65 years and older living with HIV. The CHANGE HIV study also represents the first cohort in Canada focused on this growing population, which provides an important platform for HIV and aging research. However, our findings should be interpreted in light of several limitations. First, the study sample was predominantly male, which limited our ability to assess potential sex or gender differences [69]. Second, the cohort only included older adults living with HIV who were engaged in care at participating clinics. Individuals enrolled in the study were generally well enough to attend clinic visits in person, provide informed consent, and complete lengthy study procedures. Routine engagement in care may also contribute to the relatively low levels of disability and higher levels of physical activity observed in this cohort. This pattern reflects a broader challenge in HIV research, whereby those who are most vulnerable, including individuals who are homebound, have severe physical or cognitive impairments, or reside in long-term care facilities, are often underrepresented in research [70]. As a result, the transferability of our findings to those not in regular HIV care or to the broader population aging with HIV requires careful consideration. Third, although we examined a wide range of commonly reported comorbidities, this list was not exhaustive. Future work should consider incorporating a broader range of comorbidities such as psychiatric conditions and HIV-associated neurocognitive disorder to more fully capture how they affect the physical, cognitive, and mental health in older adults living with HIV. In addition, collecting information on the severity, duration, or management (pharmacological and nonpharmacological) of each condition could provide a more precise understanding of their individual impact. From a methodological perspective, we assumed an additive relationship and assigned equal weight to all comorbidities. However, different comorbidities may result in varying types and amounts of disability in practice. Alternative approaches such as non-linear, interaction, or disease-specific effects could be adopted to assess the complexity of comorbidities to a fuller extent. Regarding directions for future research, investigators may consider incorporating other patient-reported outcome measures that capture disability experiences beyond basic ADLs and more fully reflect the multidimensional (physical, emotional, cognitive and social) nature of disability in the context of HIV [71, 72]. This line of research would also benefit from longitudinal analyses to disentangle temporal relations among comorbidity, disability, and physical activity and to clarify potential causal pathways.

## Conclusions

Drawing on the first Canadian cohort of older adults living with HIV, this study shows that dyslipidemia, hypertension, cancer, diabetes, and arthritis are among the most common comorbidities, and that complex combinations of coexisting conditions represent significant challenges in the context of aging with HIV. A greater number of comorbidities was associated with more severe disability, highlighting their cumulative impact on everyday functioning. Importantly, physical activity emerged as a protective factor that attenuated this relationship. As the population of people living with HIV continues to age, our findings underscore the need for comprehensive, person-centered health and rehabilitation services that address comorbidities associated with HIV. In addition to routine screening and management of chronic conditions, integrating accessible, tailored physical activity interventions into standard HIV care may be a pragmatic strategy to reduce disability and promote healthy aging among older adults living with HIV.

## Supplementary Information

Supplementary Material 1.

## Data Availability

The datasets generated and/or analysed during the current study are not publicly available as participants did not consent to providing public access to their data in the consent process. Data are available upon reasonable request from the corresponding author and are subject to review by the Research Ethics Board at the University of Toronto.

## Abbreviations

ADLs: Activities of Daily Living
BI: Barthel Index
CDC: Centers for Disease Control and Prevention
CHANGE HIV study: Correlates of Healthy Aging in Geriatric HIV study
EDQ: Episodic Disability Questionnaire
FAC: Functional Ambulation Classification
HIV: Human Immunodeficiency Virus
HAQ-DI: Health Assessment Questionnaire Disability Index
HDQ: HIV Disability Questionnaire
OHIP: Ontario Health Insurance Plan
RAPA: Rapid Assessment of Physical Activity
REB: Research Ethics Board
SPPB: Short Physical Performance Battery
