# Supplementary material for: The interplay of comorbidity, disability, and physical activity among older adults living with HIV: insights from the CHANGE HIV study
Source: BMC Geriatr. 2026 Jan 6;26:159. doi: 10.1186/s12877-025-06939-w (PMC12870037; doi:10.1186/s12877-025-06939-w)
Supplement: Supplementary file 1 — Supplementary Material 1. [file 12877_2025_6939_MOESM1_ESM.docx]

**Supplementary Table 1**. Cross-tabulation of individual comorbidities by physical activity level among older adults living with HIV in the CHANGE HIV Study

|  | Hypertension | | Coronary heart disease | | Congestive heart failure | | Dyslipidemia | | Stroke | | Peripheral neuropathy | | Parkinson’s disease | |
| --- | --- | --- | --- | --- | --- | --- | --- | --- | --- | --- | --- | --- | --- | --- |
| Physical activity level | Yes | No | Yes | No | Yes | No | Yes | No | Yes | No | Yes | No | Yes | No |
| Level 1: Sedentary | 13 (6) | 9 (3) | 4 (5) | 18 (4) | 0 | 22 (5) | 12 (5) | 10 (4) | 4 (18) | 18 (4) | 2 (2) | 20 (5) | 0 | 22 (4) |
| Level 2: Underactive | 13 (6) | 10 (4) | 3 (4) | 20 (5) | 1 (5) | 22 (5) | 14 (5) | 9 (4) | 0 | 23 (5) | 3 (3) | 20 (5) | 0 | 23 (5) |
| Level 3: Underactive regular light | 30 (13) | 23 (8) | 9 (11) | 44 (10) | 2 (11) | 51 (11) | 22 (8) | 31 (13) | 9 (41) | 44 (9) | 15 (17) | 38 (9) | 0 | 53 (11) |
| Level 4: Underactive regular | 45 (19) | 75 (27) | 22 (27) | 96 (23) | 3 (16) | 115 (24) | 59 (23) | 60 (24) | 3 (14) | 115 (24) | 22 (24) | 97 (23) | 2 (67) | 117 (23) |
| Level 5: Active | 131 (57) | 161 (58) | 43 (53) | 248 (58) | 13 (68) | 278 (57) | 155 (59) | 138 (56) | 6 (27) | 285 (59) | 49 (54) | 241 (58) | 1 (33) | 290 (57) |
|  | COPD | | Osteoporosis | | Arthritis | | Diabetes | | Chronic kidney disease | | Cancer | | Depression | |
| Physical activity level | Yes | No | Yes | No | Yes | No | Yes | No | Yes | No | Yes | No | Yes | No |
| Level 1: Sedentary | 5 (10) | 17 (4) | 4 (5) | 18 (4) | 3 (3) | 19 (5) | 11 (9) | 11 (3) | 7 (12) | 15 (3) | 5 (4) | 17 (5) | 5 (7) | 17 (4) |
| Level 2: Underactive | 6 (12) | 17 (4) | 3 (4) | 20 (5) | 3 (3) | 20 (5) | 9 (7) | 14 (4) | 5 (9) | 18 (4) | 6 (4) | 17 (5) | 5 (7) | 18 (4) |
| Level 3: Underactive regular light | 4 (8) | 49 (11) | 8 (10) | 45 (10) | 15 (14) | 38 (9) | 16 (13) | 37 (10) | 9 (15) | 44 (10) | 19 (13) | 34 (9) | 9 (12) | 44 (10) |
| Level 4: Underactive regular | 12 (25) | 106 (23) | 21 (27) | 99 (23) | 25 (23) | 95 (24) | 29 (24) | 90 (23) | 12 (20) | 108 (24) | 38 (27) | 81 (22) | 18 (24) | 103 (23) |
| Level 5: Active | 22 (45) | 269 (59) | 42 (54) | 250 (58) | 61 (57) | 231 (57) | 56 (46) | 236 (61) | 26 (44) | 264 (59) | 74 (52) | 217 (59) | 39 (51) | 258 (59) |

*Note*: COPD = Chronic obstructive pulmonary disease.

**Supplementary Table 2.** Hierarchical associations between comorbidity, disability, and physical activity, adjusting sequentially for contextual factors (sensitivity analyses)

|  | Outcome: severity of disability (range: 0–3) | | | |
| --- | --- | --- | --- | --- |
|  | Model 1 | Model 2 | Model 3 | Model 4 |
| Contextual factors | Unstandardized regression coefficients *Beta* [95% confidence intervals] | | | |
| Comorbidity (counts) | 0.08 [0.05, 011]*** | 0.08 [0.05, 0.10]*** | 0.06 [0.04, 0.09]*** | 0.06 [0.04, 0.09]*** |
| Physical activity level (ref: non-active†) |  |  |  |  |
| Active | –0.02 [–0.13, 0.10] | –0.00 [–0.12, 0.12] | 0.03 [–0.08, 0.14] | 0.03 [–0.08, 0.14] |
| Comorbidity × Active | –0.06 [–0.10, –0.02]** | –0.06 [–0.10, –0.02]** | –0.05 [–0.08, –0.01]** | –0.05 [–0.08, –0.01]** |
| Age (per 10-year) |  | 0.02 [–0.05, 0.10] | 0.01 [–0.06, 0.09] | 0.02 [–0.06, 0.09] |
| Sex (ref: female) |  |  |  |  |
| Male |  | –0.05 [–0.16, 0.07] | –0.03 [–0.13, 0.08] | –0.03 [–0.13, 0.08] |
| Race/Ethnicity (ref: White) |  |  |  |  |
| Black |  | 0.01 [–0.10, 0.12] | 0.01 [–0.09, 0.11] | –0.01 [–0.12, 0.09] |
| Other |  | 0.08 [–0.03, 0.18] | 0.09 [–0.00, 0.19] | 0.08 [–0.02, 0.18] |
| Education (ref: trades certificate or below) |  |  |  |  |
| College or university diploma |  | 0.02 [–0.06, 0.11] | 0.02 [–0.06, 0.10] | 0.03 [–0.05, 0.11] |
| Bachelor’s degree or higher |  | 0.04 [–0.04, 0.13] | 0.03 [–0.05, 0.10] | 0.03 [–0.05, 0.11] |
| Household income (ref: under $50,000) |  |  |  |  |
| $50,000 – $99,999 |  | –0.10 [–0.19, –0.02]* | –0.08 [–0.15, –0.00]* | –0.08 [–0.16, –0.01]* |
| More than $100,000 |  | –0.09 [–0.19, 0.02] | –0.05 [–0.14, 0.05] | –0.05 [–0.15, 0.04] |
| Marital status (ref: unpartnered) |  |  |  |  |
| In a relationship |  | –0.03 [–0.10, 0.05] | 0.01 [–0.06, 0.08] | 0.00 [–0.08, 0.08] |
| Employment (ref: retired) |  |  |  |  |
| Employed |  | –0.09 [–0.18, 0.00] | –0.06 [–0.14, 0.02] | –0.06 [–0.15, 0.02] |
| Other (volunteer, homemaker, etc.) |  | –0.07 [–0.18, 0.04] | –0.05 [–0.16, 0.05] | –0.04 [–0.15, 0.06] |
| Age group at HIV diagnosis (ref: <50 years) |  |  |  |  |
| 50 years and older |  |  | –0.01 **[**–0.09, 0.06**]** | –0.02 **[**–0.09, 0.05**]** |
| CD4^+^ nadir (per 100 cells/mm^3^) |  |  | –0.00 [–0.02, 0.02] | –0.00 [–0.02, 0.02] |
| Physical capacity (SPPB score) |  |  | –0.07 [–0.08, –0.05]*** | –0.07 [–0.08, –0.05]*** |
| Loneliness (per 10-point) |  |  | 0.03 [0.01, 0.06]* | 0.03 [–0.00, 0.06] |
| HIV stigma (per 10-point) |  |  |  | 0.04 [–0.02, 0.10] |
| Social support (per 10-point) |  |  |  | 0.00 [–0.01, 0.02] |
| Adjusted R^2^ | 0.12 [0.07, 0.17] | 0.13 [0.08, 0.19] | 0.28 [0.21, 0.34] | 0.28 [0.21, 0.35] |

*Note*: †Because only 4% of participants in the CHANGE HIV study were classified as sedentary, we performed sensitivity analyses where the non-active group included participants with RAPA physical activity levels ranging from Level 1 (sedentary) to Level 4 (underactive regular) to determine the robustness of our findings. Ref = reference group. Model 1 tested the main and interaction effects of comorbidity and physical activity on disability severity. Model 2 included personal characteristics and socioeconomic status. Model 3 included correlates in Model 2 plus HIV-specific metrics and health-related factors. Model 4 included correlates in Model 3 plus perceived stigma and social support. Unstandardized regression coefficients were reported * *p*<.05. ** *p*<.01. *** *p*<.001.

**Supplementary Figure 1.** The moderating role of physical activity in the relationship between comorbidity and disability (sensitivity analyses)


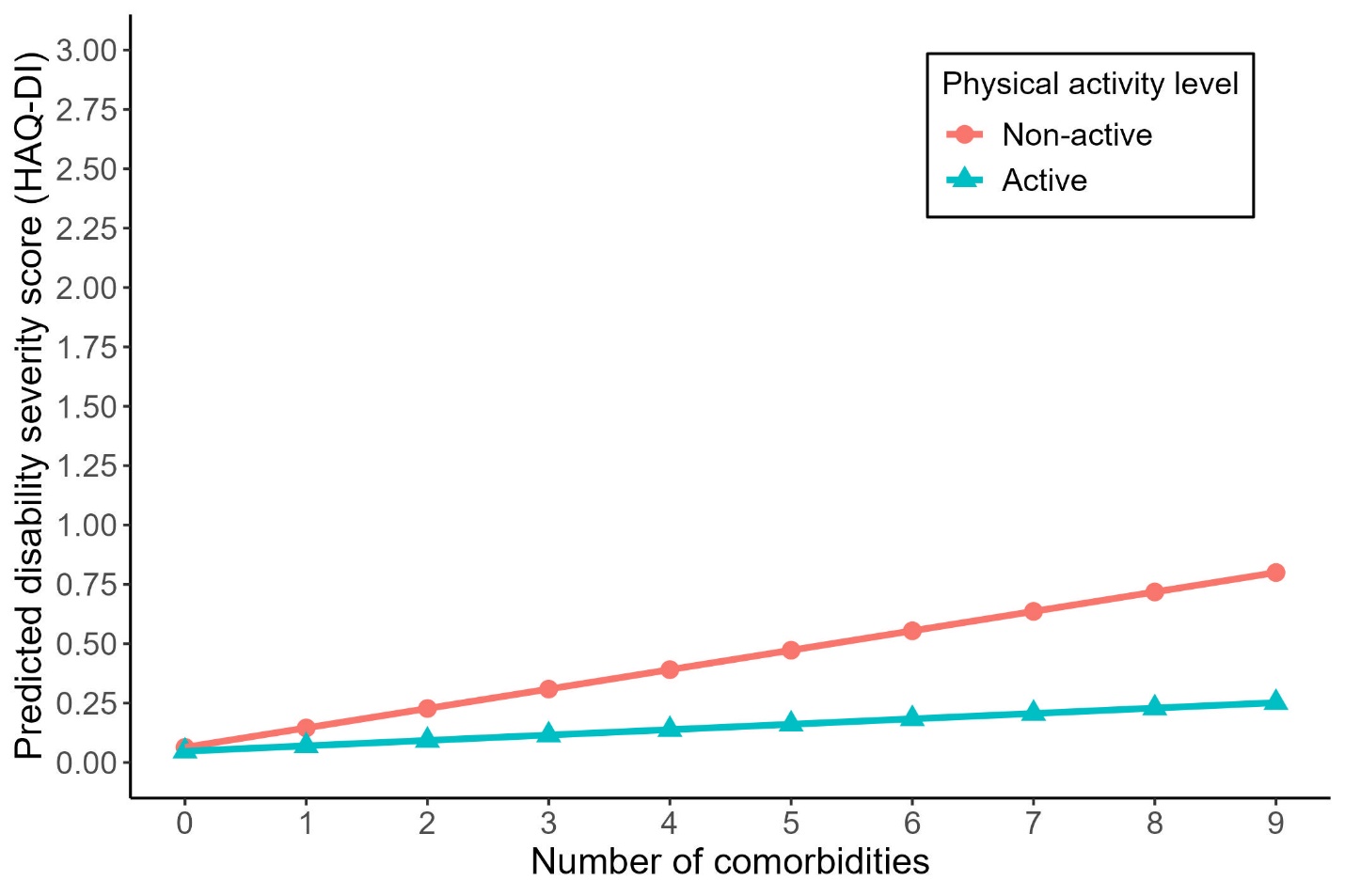


*Note*: The interaction plot illustrates the moderating role of physical activity in the base model (Model 1 in Supplementary Table 2). Compare to those who were non-active, the impact of each additional comorbidity on disability severity was less pronuced among participants who were physically active (defined as engaging in ≥30 minutes/day of moderate activity on ≥5 days/week, or ≥20 minutes/day of vigorous activity on ≥3 days/week). This pattern remains consistent after adjusting for the influence of contextual factors (Models 2–4 in Supplementary Table 2).
